# Social media popularity and election results: A study of the 2016 Taiwanese general election

**DOI:** 10.1371/journal.pone.0208190

**Published:** 2018-11-28

**Authors:** Xiaodong Zhang

**Affiliations:** Institute for the Study of Global Issues, Graduate School of Social Sciences, Hitotsubashi University, Kunitachi, Tokyo, Japan; Institute for Complex Systems, CNR, ITALY

## Abstract

This paper investigates the relationship between candidates’ online popularity and election results, as a step towards creating a model to forecast the results of Taiwanese elections even in the absence of reliable opinion polls on a district-by-district level. 253 of 354 legislative candidates of single-member districts in Taiwan’s 2016 general election had active public Facebook pages during the election period. Hypothesizing that the relative popularity of candidates’ Facebook posts will be positively related to their election results, I calculated each candidate’s Like Ratio (i.e. proportions of all likes on Facebook posts obtained by candidates in their district). In order to have a measure of online interest without the influence of subjective positivity, I similarly calculated the proportion of daily average page views for each candidate’s Wikipedia page. I ran a regression analysis, incorporating data on results of previous elections and available opinion poll data. I found the models could describe the result of the election well and reject the null hypothesis. My models successfully predicted 80% of winners in single-member districts and were effective in districts without local opinion polls with a predictive power approaching that of traditional opinion polls. The models also showed good accuracy when run on data for the 2014 Taiwanese municipal mayors election.

## Introduction

Social media played a notable role in both the 2012 general and the 2014 local elections and as well as the latest 2016 general election in Taiwan. Facebook is the most popular and well known social networking service (SNS) in Taiwan. According to reports from TWNIC and InsightXplorer, 86.3% people reported using the Internet in the previous six months, and 79.1% reported using an SNS. 99.3% of the SNS users reported owning a Facebook account compared to only 45.7% of respondents reporting ownership of a Twitter account. Few legislative candidates use Twitter for political campaigns. For example, I could not find any candidate in the Taipei 1st district in the 2016 election with a Twitter account. While presidential candidate Tsai Ing-wen does have a Twitter account, the tweets are in English and Japanese so it is clearly aimed at overseas readers. In other words, whatever the merits of using Twitter data to predict elections in other countries, Twitter data is not very useful for the Taiwanese case. However, unlike Twitter, general users’ Facebook accounts are private and hence not collectable. In this paper I therefore study the public Facebook activities of politicians rather than the private Facebook activities of voters.

Opinion polls remain the most reliable method of predicting election results. However, while opinion polls are carried out for the Taiwanese Presidential election, most single-member districts lack reliable polls by mass media or research institutes, making it hard to predict results of individual districts using traditional methods.

My research question is, therefore, ‘Can Facebook data help us forecast the results of Taiwanese elections on a district-by-district level even in the absence of reliable opinion polls?’ I will try to reject the null hypothesis that the ‘popularity of candidates on Facebook does not correlate with their vote share in the election’ using a Pearson correlation test.

In the following sections, I first review related works, then proceed to describe my methods and data collection processes. Section 4 presents my results, and the last two sections contain discussion and conclusions.

## Related work

Many papers have been written about using social media data to forecast election results [1]. In particular, researchers using Twitter data point to its remarkable power to predict real-world outcomes not only in elections [2–4] but also at the movie box office [5], on the stock market [6] and in other areas. As the result, electoral predictions using Twitter are the most common related works. For example, Burnap et al. [7] used sentiment analysis on a Twitter sample to predict the UK 2015 General Election.

However, many studies note the limitations of these researches, most notably the sample’s lack of socio-demographic controls. Gayo-Avello [8] points out the problems of using self-selected or biased samples. Jungherr et al. [9] criticize a paper by Tumasjan et al. [10] predicting 2009 German elections with Twitter data, arguing that the results were contingent on arbitrary choices by the authors.

Electoral studies using Facebook are much less numerous than those using Twitter. Williams and Gulati [11] concluded from their analysis of social media in the 2008 US Presidential nominating contests and final election that ‘Facebook support is an important additional indicator of candidate electoral success that is independent of traditional measures like expenditures, media coverage and organizing activities as represented by campaign events.’

One obvious approach to using Facebook is to count likes and friends. For example, Barclay et al. [12] found a strong positive correlation between the number of ‘Likes’ a party or its leader received on their Facebook page and their share of the popular vote in India. They also found that time had a moderating effect on the positive relationship between them. Cameron et al. [13] measured the social media popularity of candidates in the 2011 New Zealand general election by the number of ‘friends’ on Facebook and the number of ‘followers’ on Twitter. Through OLS regression models they found that the number of Facebook friends is a significant predictor of vote share. They also found that change in the number of friends over the period prior to election day is associated with vote share.

Wang [14] used an online survey to investigate Facebook usage during the 2012 Taiwanese presidential election, and confirmed the positive relationship between uses of social media and voters’ political participation.

There is also a growing body of literature about prediction using data on Internet activity from sources such as search engines and online encyclopedias. However, Lui et al. [15], having grouped races for the 2008 and 2010 congressional elections into three groups depending on whether the candidates had Google Trends records or not, concluded that Google Trends were not a good predictor of the results.

Despite these new developments, opinion polls remain the most common and reliable method of election forecasting. This paper therefore tries to overcome the limitations of social media data by combining them with Internet activity data, opinion poll data and previous election results.

## Data and methods

### 2016 Taiwanese general election

**Fig 1:**
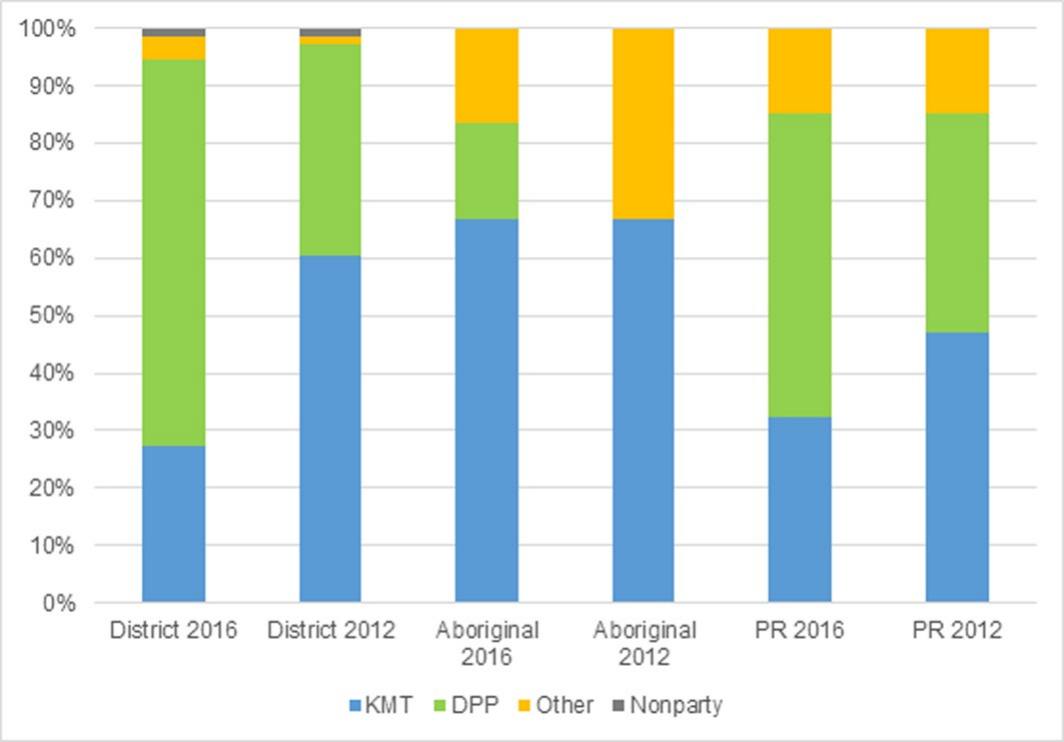
Percentage of Parliamentary Seats by parties in 2012 and 2016.

The January 2016 Taiwanese general election consisted of a presidential election and a legislative election. The legislative election part, which this paper focuses on, was made up of three parts. 354 candidates competed for 73 seats in single-member districts; 23 candidates stood for six seats of aborigines in multi-member districts; and 179 candidates were on the party lists for selection to 34 seats by proportional representation. The DPP (Democratic Progressive Party) led by Tsai Ing-wen won a resounding victory over the KMT (Kuomintang of China) which had held power since 2008. DPP won the presidential election with 56.1% of the votes and the largest winning margin since 1996. The DPP also won 68 out of 113 seats (Fig 1) in the legislative election with 45.08% of district votes and 44.04% of PR votes. For the first time a non-KMT party controlled both the presidential office and the legislative yuan.

### Data collection

In this paper I analyze social media use by candidates in the single-member districts, which account for 73 of the 113 seats in the legislature. Although the presidential election generated a large amount of social media traffic, the single-member districts provide more samples, making it better suited to statistical analysis. In addition, traditionally every party only supports one candidate in each district, making it easier to obtain variables such as result of the previous election for each party. First, I used data from the Internet to create variables showing both candidates’ social media popularity, and also measures of online interest in them without the influence of subjective positivity. I also obtained a measure of the party’s local influence (the result of previous elections in the district) and evidence from opinion polls where available. I complied with the terms of service for all the websites which I collected data, including www.facbook.com, www.wikipedia.org and db.cec.gov.tw (database of Taiwanese central election commission). As a result, our models have the following variables:

### Like ratio

Candidates in the election used Facebook in two different ways. The most common way was to have a verified page named either with the candidate’s full name, the candidate’s name plus their district or/and their party, or with a slogan (e.g. 堅持.張廖萬堅, means ‘hang in, Chang Liao Wan-chien’). These pages could both be liked and followed. In additional, posts of these pages could be seen and commented on by anyone, which means they could function as a platform for communication or as a fan club. The less common way was to create an account (sometimes verified) that could be followed, which means it was basically a private Facebook account that allowed people to follow it without being the owner’s friend. In the data I collected, these ‘followable’ accounts received far fewer likes for posts than public pages.

On 15 January 2016, I confirmed that 253 of the 354 candidates in single-member districts had active Facebook pages or followable accounts during the election period and I was able to obtain a full dataset for 222 candidates: 217 pages and five followable accounts in February 2017 (Table 1).

**Table 1:** Facebook using conditions for main parties.

Party	Candidates	Candidates with page	Candidates with followable account	Seats won
KMT	72	69	2	20
DPP	60	59	1	49
PFP^a^	6	6	0	0
MKT^b^	13	13	0	0
TSU^c^	2	2	0	0
NPP^d^	12	6	0	3
NP^e^	2	2	0	0
Green-Social^f^	11	11	0	0
Other parties	110	49	2	0
Nonparty	66	31	0	1

^a^People First Party

^b^Minkuotang

^c^Taiwan Solidarity Union

^d^New Power Party

^e^New Party

^f^Green-Social Democratic Coalition

I collected the like counts for all the pages (LPa) between 23:00 and 23:45 on 15 January 2016. At that time, I thought that collecting like counts for pages (and follower counts for accounts) would be sufficient. However, further consideration raised the problem of a possible snowball effect on number of likes of pages over time—pages might have more likes simply because they have been in existence longer. This could introduce a party bias because in general DPP candidates started using Facebook for political purposes earlier than those of the KMT. On the other hand, while likes of pages change over time, most likes of posts are registered during the first few days after the post is published. For this reason, I also collected the number of likes candidates obtained for each of their posts (LPo) published during the 30 days before the election. This second collection was carried out on 13 February 2017,

To summarize, I used two measures of likes:

1. LPa (Likes for pages): The number of likes on candidates’ Facebook pages, or the number of followers in the case of a followable account.
2. LPo (Likes for posts): The average number of likes candidate obtained for posts posted between 08:00 17 December 2015 and 08:00 16 January 2016.

I used the Python programing language and the Facebook Graph API for collecting data from pages. However, I was not able to use the Graph API to obtain data for the followable accounts—the API does not provide data about private accounts, followable or not. I therefore collected the data from these accounts manually.

**Fig 2:**
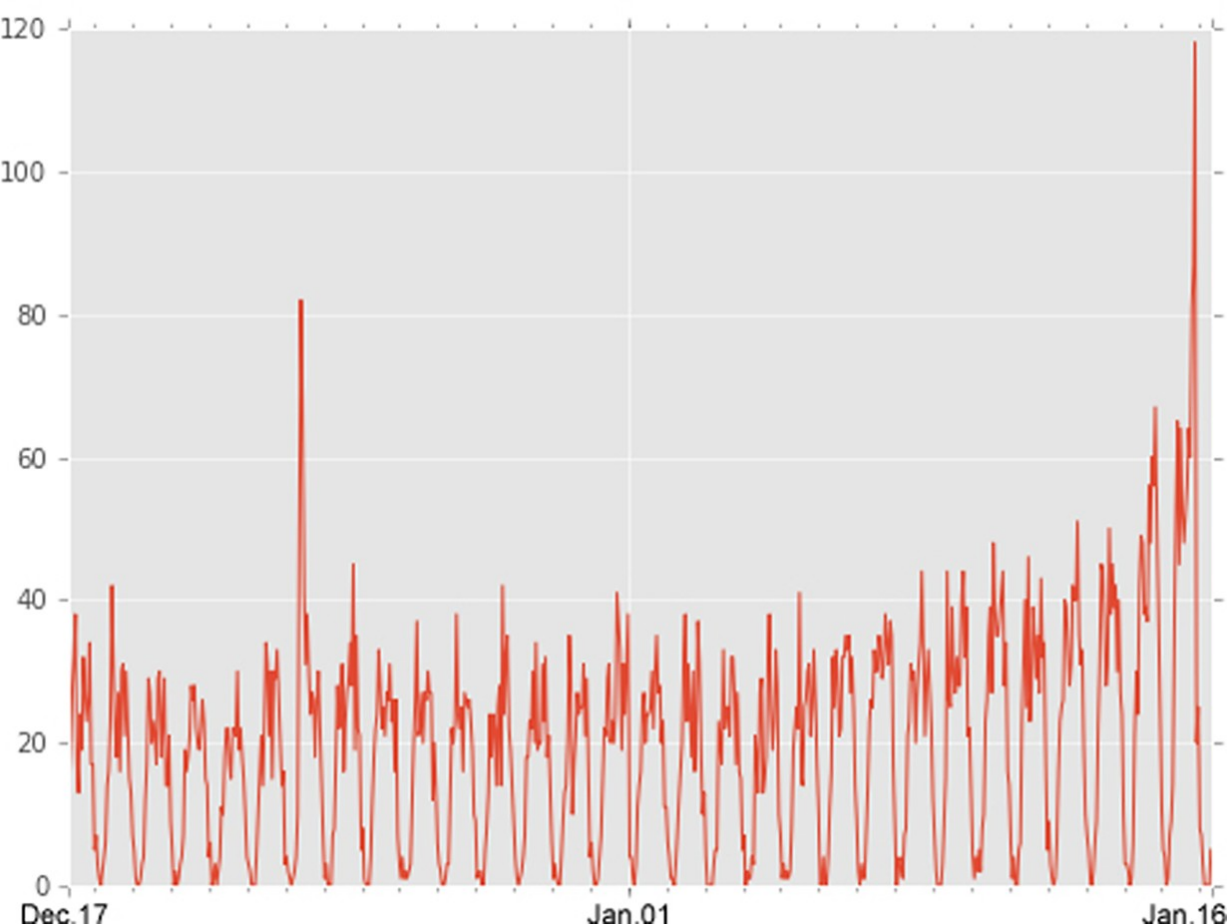
Hourly posts posted by candidates.

**Fig 3:**
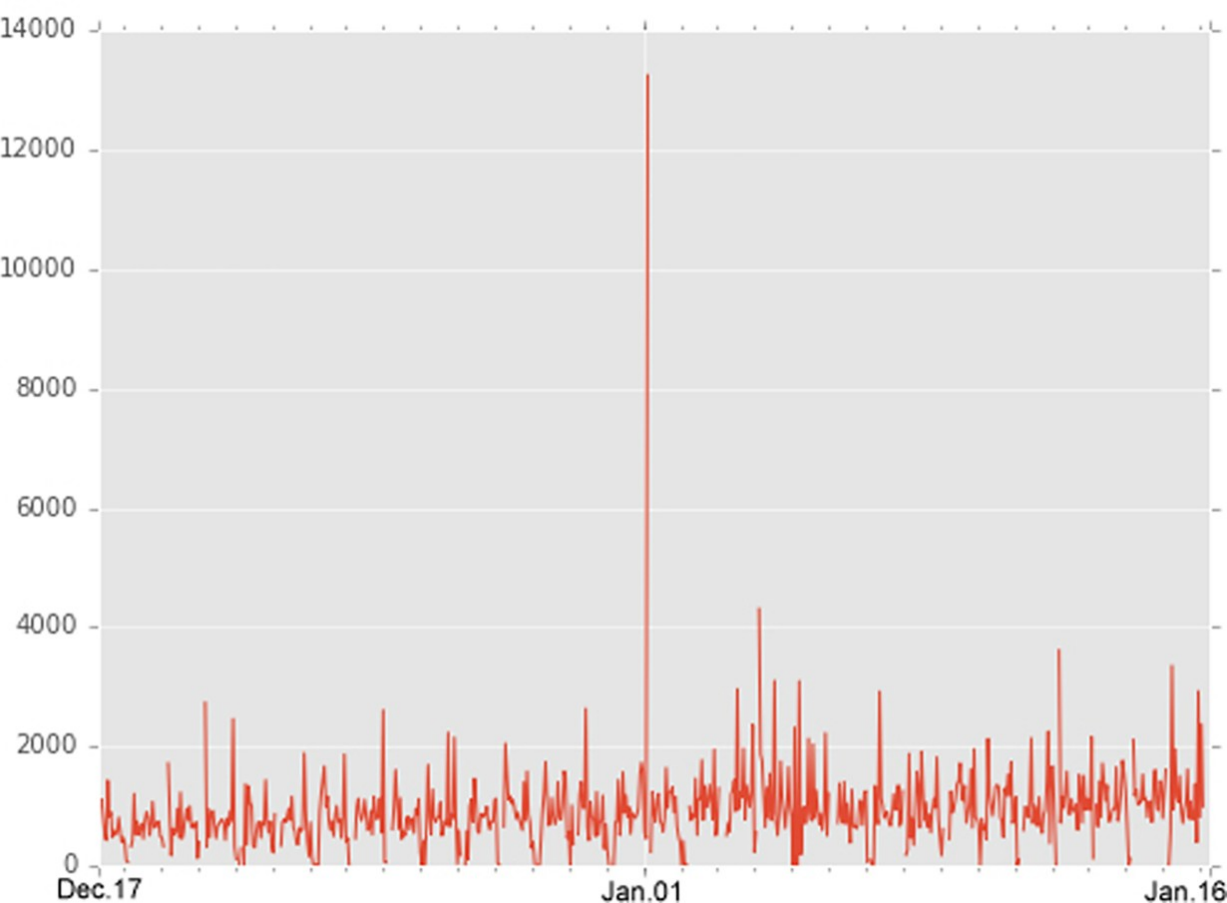
Mean likes obtained by all posts (x-axis is the created time of post).

For each of the retrieved posts, I extracted basic information including its id, date and time of creation and updating, and like count. I collected 14,096 posts from 217 pages; each post received an average of 988.63 likes (SD = 2030.74). In plus, every candidate received an average of 841.38 likes for each of their posts and the standard deviation is 1408.83. Fig 2 shows the number of posts posted by candidates hourly and Fig 3 shows the number of mean likes obtained by all posts in time order.

All main parties made active use of Facebook. The relatively traditional KMT obtained fewer likes than the other parties. Independent candidates and these from minor parties logged far behind in terms of both activity and popularity. In Table 2, Nonparty candidates supported by the DPP are included in DPP category.

**Table 2:** Data collecting results sorted by parties.

Party	Number of pages	Mean posts of each page	Mean likes obtained by each post
KMT	60	61.63	779.39
DPP	63	77.56	1466.68
Other 6 main parties	37	77.05	1224.81
Other minor parties	36	48.69	97.6
Nonparty	21	43.24	246.81

Candidates in populous urban areas are likely to receive more likes than those living in remote rural constituencies. In addition, electoral districts vary considerably in the number of voters. For example, there are 322,726 voters in New Taipei City 1st district but only 9,921 in Lianjiang County district. In order to avoid interference from these disparities, I created ‘Like Ratios’ expressing each candidate’s likes as a proportion of all likes obtained by candidates in their district. I calculated these ratios for both pages/accounts and posts.

For every candidate, I calculated the sum of Likes in the constituency, and then calculate every candidate’s *LR* (Like Ratio).

### Wiki pageviews

Users usually press ‘Like’ to express their positive attitude towards a candidate. The Like Ratio is therefore a measure of user attitude that is heavily biased towards subjective positivity. In order to have another, more neutral measure of online interest in candidates, I also collected data for the candidates’ Wikipedia pages in Traditional Chinese of Taiwan. I used Wikimedia Tool Labs to collect the data of daily average page views for the candidates’ pages between 17 December 2015 and 15 January 2016.

Then I calculate each candidate’s *WR* (Wiki Ratio) as a proportion of all candidates’ page views in the district (i.e. daily average pageviews).

### Results of previous election

I used the parties’ results in the previous (2012) election as a measure of candidates’ local influence. This is justified because Taiwanese politics is a typical two-party system, dominated by the KMT and the DPP, and because in both the 2012 and 2016 elections, all parties supported only one candidate in each single-member district.

Supporting from other parties was not rare in the 2016 election, especially in Taipei City. In order to challenge the KMT’s Pan-Blue Coalition which had controlled the majority of districts in Taipei since 1992, the DPP supported six non-DPP candidates in eight Taipei districts, including three independent candidates, one from the NPP, one from Green-Social and one from the PFP. There were a total of 13 cases of more than one party supporting a candidate, one by the KMT and 12 by the DPP. In order to deal with this, I calculated the *RoE* (Results of Last Election) as the sum of percentage of votes from each of their supporting parties earned at the previous election. In cases where the candidate stood in 2012 as an independent candidate in the district against candidates from the two major parties, but in 2016 was supported by one the major parties, I added his/her own share of the vote in the previous election to that of the party in the previous election.

### Opinion polls

While *RoE* shows the local influence four years previously, I needed a measure of popularity closer to the 2016 election. Reliable opinion polls of particular districts by mass media or research institutes were only carried out in 25 highly competitive districts. I call these opinion polls ‘local opinion polls’. For each candidate in these districts, I calculated their average poll score from the mean value for each polling organization.

For districts without local opinion polls, I used their parties’ national opinion poll results as a measure of each candidate’s popularity in the period leading up to election. I collected 14 polls from five organizations: TVBS, United Daily News, Taiwan Thinktank, Trendgo and Cross-Strait Policy Association between October 2015 and January 2016. Since opinion polling is not the major part of my model, I did not weight by organization or published date. I calculated each party’s national opinion poll score by first obtaining their mean score from each polling organization, then calculating the mean of those scores. Then, because some candidates are supported by more than one party, for each candidate in a district without local opinion polls I calculated their polling score *P* (Opinion Poll) as the sum of their supporting parties’ national opinion poll scores.

Table 3 shows opinion poll scores for the eight largest parties, the result does not include aboriginal multi-member districts.

**Table 3:** Average opinion poll results for the eight largest parties between October 2015 and January 2016.

Parties	KMT	DPP	PFP	MKT	TSU	NPP	NP	Green-Social
**Polls**	24.38%	33.02%	2.42%	0.88%	0.76%	2.15%	0.70%	0.96%
**Results**	38.71%	45.08%	1.26%	1.63%	0.82%	2.94%	0.63%	1.71%

### Data analysis

Many candidates in the legislative elections had no prospect of winning many votes. Eliminating these ‘non-competitive’ candidates could improve the accuracy of my predicting models. I therefore defined non-competitive candidates as those meeting all the following criteria:

P=0;RoE=0;LR(LPa)<3%andLPa<500;WR=0.

119 of the 354 candidates were thus defined as non-competitive. In the event, only five of those candidates received more than 3% of votes in the election. One of them, Lin Chin Kuan, won 17.67%, but this outlier can probably be explained by the small size of the electorate in the district, Lianjiang County: 17.67% means only 760 votes.

Meanwhile, 27 competitive candidates with neither data in the previous election nor opinion poll data (P = 0 and RoE = 0) received more than 3% of votes, and 15 of them received more than 5% of votes.

Some competitive candidates standing in 13 districts had Facebook data at the time of the election (i.e. LPa>0) but had deleted posts and/or their pages before I collected post data in early 2017 (i.e. (LPo = 0)). These districts (with 77 candidates) and candidates were excluded from models using *LR*(LPo).

The next step was simply to work out the Pearson correlation coefficients of the independent variables above, with vote share as the dependent variable. I set up two models: model A contains all candidates and model B includes only candidates with LR(LPo). Both these models include both competitive and non-competitive candidates.

Summarizing Table 4, I find a significant correlation between vote share and all my independent variables. The Internet variables *LR* and *WR* both show strong correlations, although both are still lower than *RoE* and *P*. We can successfully reject the null hypothesis that popularity of candidates on Facebook does not correlate with their vote share in the election.

**Table 4:** Pearson correlation coefficients for Models A and B (DV = vote share).

Ind. Variable	Model A	Model B
*LR*(LPa)	.790***	
*WR*	.771***	.758***
*RoE*	.942***	.941***
*P*	.946***	.950***
*LR*(LPo)		.825***
N	354	277

* *p*<0.05.

** *p*<0.01.

*** *p*<0.001.

Having thus confirmed the relationship between my independent variables and the election results, I set up six controlled models (numbers 0–1 thru 0–6) which reduce the number of independent variables in order to discover how our models perform with only Internet variables (*LR* and *WR*) or only non-Internet variables (*RoE* and *P*). As shown in Table 5, the four controlled models without Internet data perform well, while the two models with *LR* and *WR* also make sense but can only explain less than 60% of the variability of the response data.

**Table 5:** Regression results for Models 0–1 thru 0–6.

Ind. Variable	Unstandardized Coefficients		Standardized Coefficients	t	Sig.	VIF
	B	Std. Error	Beta			
**Model 0–1 –all candidates.** **(N = 354, Adjusted R**^**2**^ = .887*** **Sig. = .000)**						
(Constant)	.032***	.005		6.089	.000	
*RoE*	.887***	.017	.942	52.571	.000	1.000
**Model 0–2 –without non-competitive candidates.** **(N = 235, Adjusted R**^**2**^ = .880*** **Sig. = .000)**						
(Constant)	.038***	.008		4.675	.000	
*RoE*	.423***	.048	.463	8.869	.000	5.315
*P*	.780***	.081	.500	9.577	.000	5.315
**Model 0–3 –using LPa, without non-competitive candidates.** **(N = 235, Adjusted R**^**2**^ = .561*** **Sig. = .000)**						
(Constant)	.107***	.015		7.205	.000	
*LR(*LPa)	.343***	.046	.435	7.475	.000	1.808
*WR*	.295***	.044	.387	6.652	.000	1.808
**Model 0–4 –using LPo, without non-competitive candidates.** **(N = 185, Adjusted R**^**2**^ = .586*** **Sig. = .000)**						
(Constant)	.114***	.016		6.924	.000	
*LR*(LPo)	.422***	.049	.549	8.628	.000	1.797
*WR*	.213***	.048	.285	4.484	.000	1.797
**Model 0–5 –only includes competitive candidates in districts without local opinion polls.** **(N = 142, Adjusted R**^**2**^ = .857*** **Sig. = .000)**						
(Constant)	.035**	.013		2.821	.005	
*RoE*	.443***	.067	.472	6.638	.000	5.003
*P*	.793***	.117	.480	6.755	.000	5.003
**Model 0–6 –only contains candidates in districts with local opinion polls.** **(N = 139, Adjusted R**^**2**^ = .918*** **Sig. = .000)**						
(Constant)	.028***	.006		4.420	.000	
*P*	1.401***	.036	.959	39.411	.000	1.000

* *p*<0.05.

** *p*<0.01.

*** *p*<0.001.

Next I create the main models using my methodology. Table 6 summarizes the results of three regression models using LPa. Model 1 contains data for all 354 candidates of single member districts, while Model 2 only includes the 235 competitive candidates. In order to simulate the situation of a total absence of local opinion polls, I created Model 3, which additionally excluded candidates in districts with local opinion polls.

**Table 6:** Regression results for Models with LPa.

Ind. Variable	Unstandardized Coefficients		Standardized Coefficients	t	Sig.	VIF
	B	Std. Error	Beta			
**Model 1 –using LPa, for all candidates.** **(N = 354, Adjusted R**^**2**^ = .950*** **Sig. = .000)**						
(Constant)	.013***	.004		3.572	.000	
*LR*(LPa)	.131***	.017	.156	7.866	.000	2.789
*WR*	.103***	.016	.126	6.497	.000	2.655
*RoE*	.406***	.032	.431	12.893	.000	7.926
*P*	.543***	.056	.343	9.666	.000	8.908
**Model 2 –using LPa, excluding non-competitive candidates.** **(N = 235, Adjusted R**^**2**^ = .924*** **Sig. = .000)**						
(Constant)	.016*	.007		2.361	.019	
*LR*(LPa)	.129***	.020	.164	6.388	.000	2.033
*WR*	.102***	.019	.134	5.245	.000	1.994
*RoE*	.405***	.038	.442	10.631	.000	5.327
*P*	.538***	.068	.345	7.905	.000	5.858
**Model 3 –using LPa, excluding non-competitive candidates and candidates in districts with local opinion polls.** **(N = 142, Adjusted R**^**2**^ = .915*** **Sig. = .000)**						
(Constant)	.014	.010		1.377	.171	
*LR*(LPa)	.129***	.026	.170	4.943	.000	1.969
*WR*	.130***	.028	.173	4.687	.000	2.264
*RoE*	.403***	.052	.429	7.772	.000	5.057
*P*	.522***	.095	.316	5.502	.000	5.470

* *p*<0.05.

** *p*<0.01.

*** *p*<0.001.

As Table 6 shows, all three models are able to explain at least 91% variability of the response data around its mean. In addition, all independent variables in our models have extremely low probability values, which proves they are significant predictors. As a matter of course, local influence and party popularity are more significant. Meanwhile, as I expected, because of the lack of local opinion polls in Model 3, predictors from Internet (*LR*(LPa) and *WR*) play more important roles than in Models 1 and 2.

I then created Models 4 thru 6, which were the same as Models 1 thru 3 but used *LR*(LPo) instead of *LR*(LPa) as the explanatory variable; the results are shown in Table 7. The results are similar but coefficients for *LR*(LPo) are slightly higher than those for *LR*(LPa).

**Table 7:** Regression results for Models with LPo.

Ind. Variable	Unstandardized Coefficients		Standardized Coefficients	t	Sig.	VIF
	B	Std. Error	Beta			
**Model 4 –using LPo, for all candidates with *LR*(LPo)** **(N = 277, Adjusted R**^**2**^ = .951*** **Sig. = .000)**						
(Constant)	.012**	.004		2.992	.003	
*LR*(LPo)	.133***	.020	.160	6.785	.000	3.176
*WR*	.099***	.017	.121	5.709	.000	2.543
*RoE*	.393***	.035	.410	11.085	.000	7.794
*P*	.575***	.064	.360	8.982	.000	9.159
**Model 5 –using LPo, for all competitive candidates with *LR*(LPo)** **(N = 185, Adjusted R**^**2**^ = .920*** **Sig. = .000)**						
(Constant)	.017*	.008		2.070	.040	
*LR*(LPo)	.132***	.024	.172	5.493	.000	2.247
*WR*	.096***	.021	.129	4.479	.000	1.894
*RoE*	.390***	.043	.419	8.977	.000	5.003
*P*	.570***	.079	.361	7.222	.000	5.734
**Model 6 –using LPo, for all competitive candidates with *LR*(LPo) in districts without local opinion polls.** **(N = 123, Adjusted R**^**2**^ = .915*** **Sig. = .000)**						
(Constant)	.016	.011		1.509	.134	
*LR*(LPo)	.139***	.031	.181	4.491	.000	2.338
*WR*	.128***	.028	.171	4.556	.000	2.036
*RoE*	.374***	.060	.388	6.223	.000	5.590
*P*	.543***	.109	.330	4.988	.000	6.276

* *p*<0.05.

** *p*<0.01.

*** *p*<0.001.

## Results

All six models I created were able to describe the results of the election well.

Comparing the six main models with the controlled models 0–1 thru 0–6, the values of adjusted R^2^ were approximately 0.06 higher than the controlled models without Internet predictors. As a further step, I checked the ANOVA results for change in R^2^ between my models and the equivalent control models. Set 1 compares two models for all competitive candidates in order to ascertain the value of Internet predictors, while Set 2 focuses on the function of non-Internet variables when facing a ‘predict with social media’ model. The R^2^ change is 0.044 in Set 1 and 0.331 in Set 2. These two numbers from Table 8 show that while predictors from Facebook and Wikipedia help to improve our model’s accuracy, it is necessary for them to use them in combination with real-world predictors.

**Table 8:** Change statistics.

Model	R Square Change	F Change	df1	df2	Sig. F Change
**Set 1 –using LPa, for all competitive candidates**					
Model 0–2	.881	859.505	2	232	.000
Model 2	.044	67.799	2	230	.000
**Set 2 –using LPo, for all competitive candidates with *LR*(LPo)**					
Model 0–4	.591	131.248	2	182	.000
Model 5	.331	380.243	2	180	.000

Besides the better explainability obtained by adding Internet predictors, I also found the value of the constant reduced by about half. This makes sense, because a non-competitive candidate with all predictor values of 0 is very unlikely to win more than 3% of votes. Thus, using additional predictors from Facebook and Wikipedia definitely improves the explanatory power of the models.

### Model evaluation

I used Mean Absolute Error (MAE) as the measure of error in our predictive forecasting models. Previous research using Twitter and Facebook data has varying reports about measures of deviation. As Bermingham and Smeaton [16] note, the observed MAE can be very low [10] or even more than 17% [8]. I calculated the MAE of all our six models and two representative controlled models, Model 0–2 as a case without Internet features and Model 0–6 as the ‘classic’ model using only local opinion poll data. As a reference, I also list the values of another popular measure, RMSE.

Table 9 shows that the observed error of our models varies from 3.31% to 4.82%, which I think is acceptably low. Similarly to the results of the regression analysis, LPo has a very slight advantage over LPa, the models using *LR*(LPo) are approximately 0.1% lower in MAE.

**Table 9:** Errors of models.

Model	N	MAE	RMSE
Model 0–2	235	0.0575	0.0768
Model 0–6	139	0.0411	0.0598
Model 1	354	0.0331	0.0507
Model 2	235	0.0455	0.0609
Model 3	142	0.0482	0.0651
Model 4	277	0.0326	0.0505
Model 5	185	0.0455	0.0614
Model 6	123	0.0464	0.0641

Observing the controlled models, I found the MAE of Model 0–2 is 5.75%, much higher than any of our models. Together with the 4.4% increase in explanatory power (see Table 8), this further underlines the value of additional predictors from the Internet. More importantly, the error of Model 6 is parked at just 4.64%, while the result of Model 0–6, a model containing only local opinion polls (i.e. a very traditional predictive model) is 4.11%. With only 0.5% better mean error, I conclude that my models can be used to predict election results in districts without local opinion polls with a predictive power approaching that of traditional opinion polls. To answer our research question, we can create a model to forecast the results of Taiwanese elections even in the absence of reliable local opinion polls in this case.

### Performance of winner prediction

In a single-member district election, a loss with 48% of the votes is still a loss. In evaluating our models, therefore, it is important to test how many winners are actually predicted.

The results shown in Table 10 again supported our previous findings. All six models showing good accuracy, LPo performs very slightly better than LPa, and we can predict outcomes in districts without local opinion polls. It is quite impressive that the accuracy of our models is over 10% higher than local opinion polls from mass media (Model 0–6).

**Table 10:** Successfully predicted districts for each model.

Model	Success	Failure	Accuracy
Model 0–2	55	18	75.34%
Model 0–6	17	8	68%
Model 1	58	15	79.45%
Model 2	58	15	79.45%
Model 3	41	7	85.42%
Model 4	48	12	80%
Model 5	48	12	80%
Model 6	35	7	83.33%

### Applying the models to other elections

The best way to check the predictive power of a model is to apply it to forecast a real election in the future. Therefore, I should at least do a simulation of how my models work in another election. I selected 2014 Taiwanese Municipal Mayors Election as my test subject. Since these elections in Taiwan are almost always two-horse races, so I only collected Facebook data for the two main candidates in each race.

I collected relevant data for the four independent variables for all candidates in the six municipalities, except the LPa for eight minor candidates. In addition, because candidates in municipal mayoral elections tend to be more famous than legislative candidates and minor candidates are more likely to have a Wikipedia page, I lifted the lower limit of WR for a competitive candidate to 3%. The LPo was collected on 7 May 2017. Since local opinion polls were carried out for all municipalities, I applied Models 2 and 5.

The results in Tables 11 and 12 show the effectiveness of my predictive models. My models successfully predicted the other five winners. Even for the sixth race, in which Cheng Wen-tsan won a totally unexpected victory in Taoyuan City, my result was far closer than most available polls and actually better than all the polls I collected. The MAE values remain low at 5.31% and 5.19% for Models 2 and 5 respectively, and both of them are lower than the simple linear regression model using opinion polls.

**Table 11:** Results of applying Models 2 and 5 to the 2014 Taiwanese municipal mayors election.

Candidate	District	Party	*RoE*	*P*	PRE(LPa)	PRE(LPo)	Vote Share
Chao Yen-ching	Taipei	n/a	0.00%	0.15%	n/a	2.71%	1.06%
Neil Peng	Taipei	n/a	0.00%	2.02%	n/a	5.20%	0.54%
Sean Lien	Taipei	KMT	55.64%	29.29%	46.37%	46.48%	40.82%
Ko Wen-je	Taipei	DPP	43.81%	43.40%	55.56%	56.29%	57.15%
Chen Chu	Kaohsiung	DPP	52.80%	55.55%	70.73%	71.78%	68.08%
Yang Chiu-hsing	Kaohsiung	KMT	47.20%	17.58%	35.41%	35.10%	30.89%
Yu Shyi-kun	NTC	DPP	47.39%	27.33%	40.25%	40.34%	48.78%
Eric Chu	NTC	KMT	52.61%	47.24%	66.65%	67.35%	50.06%
Jason Hu	Taichung	KMT	51.12%	31.40%	47.90%	48.06%	42.93%
Lin Chia-lung	Taichung	DPP	48.88%	43.33%	59.10%	59.74%	57.06%
Lai Ching-te	Tainan	DPP	60.41%	63.40%	79.04%	80.17%	72.89%
Huang Hsiu-shuang	Tainan	KMT	39.59%	16.27%	30.62%	30.44%	27.10%
Cheng Wen-tsan	Taoyuan	DPP	45.69%	25.37%	50.29%	50.56%	51.00%
Wu Chih-yang	Taoyuan	KMT	52.22%	47.47%	54.85%	55.34%	47.96%

**Table 12:** Errors of models.

Model	N	MAE	RMSE
Model 2	12	0.0531	0.0668
Model 5	14	0.0519	0.0661
Regression Model with P	14	0.0595	0.0752

Although one test in an election with only six seats is not enough to confirm the predictive power, this is a promising first confirmation of the accuracy of my forecast models.

## Discussion

While the results suggest I have achieved the majority of my research goals, some concerns remain.

### Does excluding non-competitive candidates really help?

I excluded candidates with very low status in all predictors for the sake of better accuracy in some of my models, and I confirmed that almost none of these excluded candidates won many votes. However, the results of the analysis do not completely support this step: the effects of Internet predictors become slightly stronger but the R2 and error get a little worse. In other words, excluding non-competitive candidates might not make much difference. In order to check the effect of excluding non-competitive candidates, I applied models including and excluding non-competitive candidates to datasets containing only competitive candidates. Table 13 confirms this suspicion. Models 1 and 4, which both all include non-competitive candidates, had 0.02% and 0.03% lower mean errors than Models 2 and 5 respectively. In other words, excluding non-competitive candidates had no effect on the performance of the models. At least in this case, therefore, it is not necessary to exclude non-competitive candidates and conversely the model can also be applied to only competitive candidates. I should observe the effects of excluding and including non-competitive candidates when applying these models to other elections in the future.

**Table 13:** Error check.

Model	N	MAE	RMSE
**Dataset 1 –all competitive candidates**			
Model 1	235	0.0453	0.0610
Model 2	235	0.0455	0.0609
**Dataset 2 –all competitive candidates with LPo**			
Model 4	185	0.0452	0.0615
Model 5	185	0.0455	0.0614

### Which model to select?

Which of the six models should we use in predicting election outcomes? As noted above, excluding non-competitive candidates did not make a noticeable difference to predictive power. Furthermore, while the models using LPo performed slightly better than those using LPa and I subjectively think they can better reflect popularity during a certain period, results and predicted values varied little between the LPa and the LPo models. Assuming that data for both LPa and LPo are available, in cases where we have partial or complete local opinion polls we should set Model 4 as the main model. In the absence of local opinion polls we should use Model 6. The other models still can be used as references, and further adjustment of model selection should be done during the future use.

### Demographic issues

As in all research that uses social media to predict real-world outcomes, demographic factors are a potential limitation. Social media use is widespread among teens, but only Taiwanese citizens over 20 years old can vote. On the other hand, social media use is much lower among older people. In addition, voters living outside a candidate’s district may like their Facebook page and posts; this happens especially in the case of famous candidates. Another possible concern in this project is the participation of Facebook users from the mainland. This phenomenon attracted people’s attention because of massive negative comments on the page of the current leader of Taiwan, Tsai ing-wen, in 2016. Because of these issues, the demographics of Facebook users who pressed like are probably quite different from the demographics of Taiwanese voters.

There is some good news, however. First, Taiwan is moving to lower the voting age to 18, bringing more Facebook users into the electorate. Second, from personal observation, most users from the mainland comment only on the pages of the most famous politicians, and not those of the candidates in the legislative and local elections under discussion here. Plus, mainland users are more likely to leave negative comments than to press ‘Like’.

While no opinion poll can obtain a perfectly random sample of the electorate, I tried to reduce demographic bias by including traditional opinion poll results (and also previous election results) in my models.

### Do the predictions get better as the election approaches?

The existing data allow us to make clear whether predictions get better as the election approaches. I created two new sets of data contain two online predictors *WR* and *LR*(LPo). The first set combines LPo for these posts created between 08:00 17 December 2015 and 08:00 9 January 2016, and Wiki viewing data between 17 Dec and 9 Jan. (i.e. up until one week prior to election day) The second set combines data from 17 Dec and 2 Jan. (i.e. up until two weeks prior to election day) I checked these earlier data in two ways: using them with existing data of *P* and *RoE* to create new models; and adopting these data in our main model, Model 4.

As Tables 14 and 15 show, predictions do indeed get better as the election approaches, but only gradually. In addition, our main Model performs better than the two new models, even using the datasets used to build the new models. However, our datasets of up until two weeks and one week before election day cover shorter periods and therefore contain far fewer samples than the full dataset; future studies should start to collect data further in advance of election day in order to have datasets of similar length before the dates of prediction.

**Table 14:** Regression results for models with earlier data.

Ind. Variable	Unstandardized Coefficients		Standardized Coefficients	t	Sig.	VIF
	B	Std. Error	Beta			
**Model 4–1 –using *LR*(LPo) and *WR* up to one week prior to election day** **(N = 277, Adjusted R**^**2**^ = .949*** **Sig. = .000)**						
(Constant)	.013**	.004		3.01	.003	
*LR*(LPo)	.102***	.018	.126	5.732	.000	2.633
*WR*	.116***	.016	.144	7.080	.000	2.266
*RoE*	.391***	.036	.408	10.815	.000	7.782
*P*	.600***	.065	.376	9.215	.000	9.068
**Model 4–2 –using *LR*(LPo) and *WR* up to two weeks prior to election day** **(N = 277, Adjusted R**^**2**^ = .950*** **Sig. = .000)**						
(Constant)	.013**	.004		3.104	.002	
*LR*(LPo)	.092***	.017	.115	5.339	.000	2.542
*WR*	.126***	.017	.156	7.455	.000	2.416
*RoE*	.381***	.036	.398	10.568	.000	7.795
*P*	.612***	.064	.384	9.509	.000	8.939

* *p*<0.05.

** *p*<0.01.

*** *p*<0.001.

**Table 15:** Error check with earlier data.

Model	N	MAE	RMSE
**Dataset 1 –earlier data in new models**			
Model 4–1	277	0.0337	0.0515
Model 4–2	277	0.0341	0.0514
**Dataset 2 –earlier data in Model 4**			
One Week	277	0.0334	0.0518
Two Week	277	0.0337	0.052

## Conclusion

My findings suggest that social media can help us forecast the results of Taiwanese elections on a district-by-district level even in the absence of local opinion polls. This supports other research suggesting that social media can be a powerful indicator when predicting election results, especially when used in combination with ‘real-world’ predictors.

Furthermore, more likes does mean more votes in our case: electoral campaigns on Facebook were found to be effective. Hence, candidates in Taiwan would be well advised to invest more attention in social media such as Facebook. Actually it was already widely noticed that popular politicians with verified public fan pages had huge numbers of likes on their pages and posts, particularly when compared to the number of voters in their constituency and the population of the whole island. More than that, the number of likes is still increasing at a remarkable speed: the number of likes for many online star politicians’ pages doubled in the year between our two data collections.

I am looking forward to applying my model to the 2018 Taiwanese local election and collecting time-series data in advance. I expect to confirm the effectiveness of my models and try to improve the accuracy and applicability by increasing the quantity of data and by introducing weighting methods.

Other topics for further research include a network analysis of the interactions on Facebook among and between candidates and voters. It may also be that the kind of campaign affects the number of likes. Finally, content analysis of the posts and comments could also reveal more about the dynamics of voters’ engagement with candidates.

## Supporting information

S1 File Data used for models. (CSV)

S2 File Data collected from Facebook. (CSV)

## References

[1] Anstead N, O’Loughlin B. Social Media Analysis and Public Opinion: The 2010 UK General Election. Journal of Computer-Mediated Communication. 2015;20(2):204–20.

[2] Chung JE, Mustafaraj E. Can Collective Sentiment Expressed on Twitter Predict Political Elections? In San Francisco, CA, USA; 2011 Available from: http://cs.wellesley.edu/~eni/papers/chung_aaai11.pdf

[3] DiGrazia D, McKelvey K, Bollen J, Rojas F. More Tweet, More Votes: Social Media as a Quantitative Indicator of Political Behavior. PLoS ONE. 2013;8(11). 10.1371/journal.pone.0079449 PMC3842288 24312181

[4] Sang ETK, Bos J. Predicting the 2011 Dutch Senate Election Results with Twitter. In Avignon, France; 2012 Available from: https://ifarm.nl/erikt/papers/sasn2012.pdf

[5] Asur S, Huberman BA. Predicting the Future with Social Media. In Toronto, Canada; 2010 p. 492–9.

[6] Bollen J, Mao H, Zeng X. Twitter Mood Predicts the Stock Market. Journal of Computational Science. 2011;2(1):1–8.

[7] Burnap P, Gibson R, Sloan L, Southern R, Williams M. 140 characters to victory?: Using Twitter to predict the UK 2015 General Election. Electoral Studies. 2016;41:230–3.

[8] Gayo-avello D. ‘I wanted to predicti elections with twitter and all I got was this lousy paper’ A balanced survey on election prediction using twitter data [Internet]. 2012 Available from: https://arxiv.org/pdf/1204.6441.pdf

[9] Jungherr A, Jurgens P, Schoen H. Why the Pirate Party Won the German Election of 2009 or The Trouble With Predictions: A Response to Tumasjan, A., Sprenger, T. O., Sander, P. G., & Welpe, I. M. ‘Predicting Elections With Twitter: What 140 Characters Reveal About Political Sentiment.’ Social Science Computer Review. 2012;30(2):229–34.

[10] Tumasjan A, Sprenger TO, Sandner PG, Welpe IM. Election forecasts with Twitter: How 140 characters reflect the political landscape. Social Science Computer Review. 2011;29(4):402–18.

[11] Williams CB, Gulati G. What is a social network worth? Facebook and vote share in the 2008 presidential primaries. In Boston, MA, USA; 2008 Available from: http://citeseerx.ist.psu.edu/viewdoc/download?doi=10.1.1.471.7745&rep=rep1&type=pdf

[12] Barclay FP, Pichandy C, Venkat A, Sudhakaran S. India 2014: Facebook ‘Like’ as a Predictor of Election Outcomes. Asian Journal of Political Science. 2015;23(2):134–60.

[13] Cameron MP, Barret P, Stewardson B. Can Social Media Predict Election Results? Evidence From New Zealand. Journal of Political Marketing. 2016;15(4):416–32.

[14] Wang T. ‘Facebook Election?’ The Impact of Social Media on Political Participation in Taiwan’s 2012 Presidential Election. Soochow Journal of Political Science. 2013;31(1):1–52.

[15] Lui C, Metaxas PT, Mustafaraj E. On the predictability of the US elections through search volume activity. In Avila, Spain; 2011 Available from: http://cs.wellesley.edu/%7Epmetaxas/e-Society-2011-GTrends-Predictions.pdf

[16] Bermingham A, Smeaton AF. On Using Twitter to Monitor Political Sentiment and Predict Election Results. In Chiang Mai, Thailand; 2011.

